# Unexpected favorable outcome to sintilimab plus bevacizumab in an *EGFR*‐mutated non‐small cell lung cancer patient: A case report

**DOI:** 10.1111/1759-7714.13569

**Published:** 2020-07-13

**Authors:** Yiruo Zhang, Mei Zhao, Shuliang Cao, Xuchao Zhang, Yingying Du

**Affiliations:** ^1^ Department of Oncology The First Affiliated Hospital of Anhui Medical Universtiy Hefei China; ^2^ Burning Rock Biotech Guangzhou China; ^3^ Guangdong Lung Cancer Institute, Guangdong Provincial Key Laboratory of Translational Medicine in Lung Cancer, Medical Research Center, Cancer Center, Guangdong Provincial People's Hospital & Guangdong Academy of Medical Sciences Guangzhou China

**Keywords:** *EGFR*‐mutant, NSCLC, PD‐1, resistance, sintilimab

## Abstract

A 53‐year‐old man diagnosed with disease stage IIIB pulmonary adenocarcinoma underwent chemotherapy and radiotherapy in the first‐line setting. After disease progression, he received targeted therapy because of subsequent detection of *EGFR* exon 19 del mutation. Following an increase in his adrenal metastases, a combination of immunotherapy and antiangiogenic therapy (sintilimab plus bevacizumab) was commenced. After one month, imaging showed that the adrenal metastases had shrunk and a progression‐free survival (PFS) of 6.0 months was achieved. In this case, we showed that the PD1 inhibitor sintilimab plus bevacizumab was effective in a refactory advanced *EGFR*‐mutant NSCLC with positive PD‐L1 expression.

**Key points:**

Our case report provides clinical evidence of the durable response of a patient with advanced EGFR‐mutant lung adenocarcinoma to a combination of immunotherapy and anti‐angiogenic agent, sintilimab and bevacizumab, as subsequent‐line therapy. Sintilimab and bevacizumab combination therapy was well‐tolerated and effective, resulting in dramatic tumor reduction and improvement in clinical symptoms.

## Graphical abstract

Our case reports an advanced EGFR‐mutant lung adenocarcinoma patient achieved PR and a PFS of 6 months to a combination of immunotherapy and anti‐angiogenic agent, sintilimab and bevacizumab, as subsequent‐line therapy. Sintilimab and bevacizumab combination therapy was well‐tolerated and effective, resulting in dramatic tumor reduction and improvement in clinical symptoms.

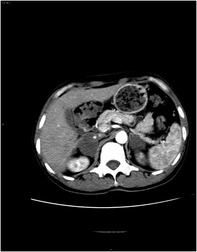

## Introduction

Lung cancer continues to be the most common malignant tumor and the leading cause of cancer death worldwide. Despite the discovery of *EGFR* (epidermal growth factor receptor) gene alterations and development of targeted therapies which has revolutionized the treatment of NSCLC, the mortality of lung cancer has continued to increase over time.^1^, ^2^ More importantly, most of patients will inevitably experience acquired resistance within less than one year.^3^

On the other hand, there has been some recent breakthroughs in cancer immunotherapy for the treatment of solid tumor patients. The activation of PD‐1 (programmed cell death protein‐1)/PD‐L1 (programmed death ligand‐1) pathway can prevent tumors from immune surveillance. Nivolumab and pembrolizumab as anti‐PD‐1 antibody and atezolizumab as PD‐L1 antibody, the immune checkpoint inhibitor (ICI), have proved their efficacy and safety in patients with pretreated advanced NSCLC in clinical trials.^4^, ^5^, ^6^ However, one of the greatest challenges is that patients harboring *EGFR* mutations may have very little opportunity to benefit from immunotherapy.^5^

Here, we report a patient with advanced NSCLC where chemotherapy and *EGFR* target therapy failed and who was administered bevacizumab in combination with sintilimab, a new anti‐PD‐1 antibody, which has shown antitumor effects and tolerability in preclinical in vitro and phase I clinical trials. The patient in this study achieved a partial response (PR) with a progression‐free survival (PFS) of six months.

### Case report

**Figure 1:**
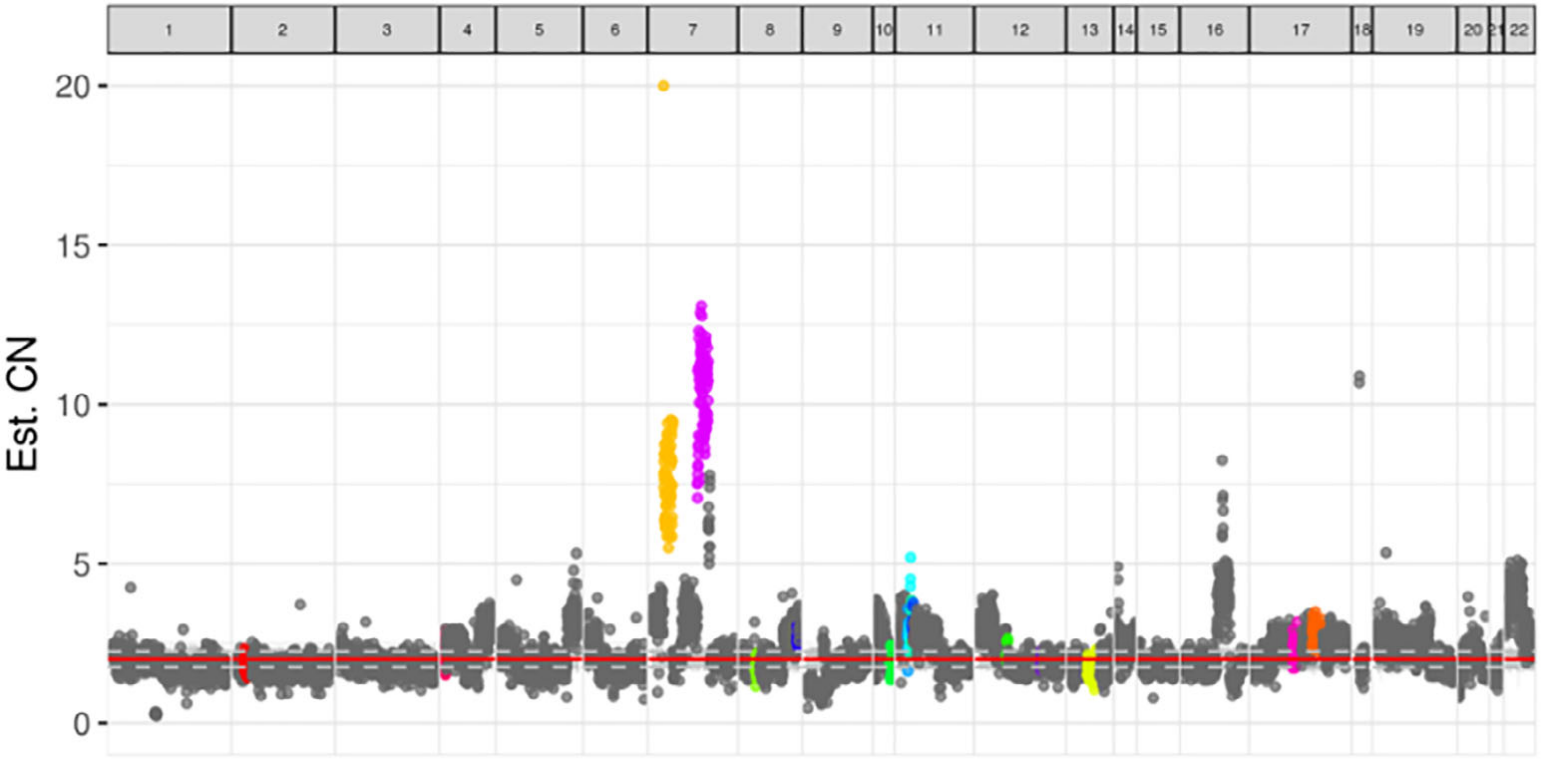
Distribution of gene copy number (purple blot: MET gene amplification). (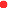) ALK; (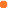) BRCA1; (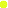) BRCA2; (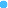) CCND1; (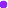) CDK4; (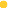) EGFR; (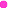) ERBB2; (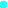) FGF19; (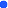) FGF3; (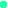) FGF4; (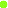) FGFR1; (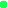) FGFR2; (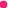) FGFR3; (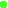) KRAS; (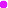) MET; (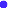) MYC; (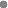) others.

**Figure 2:**
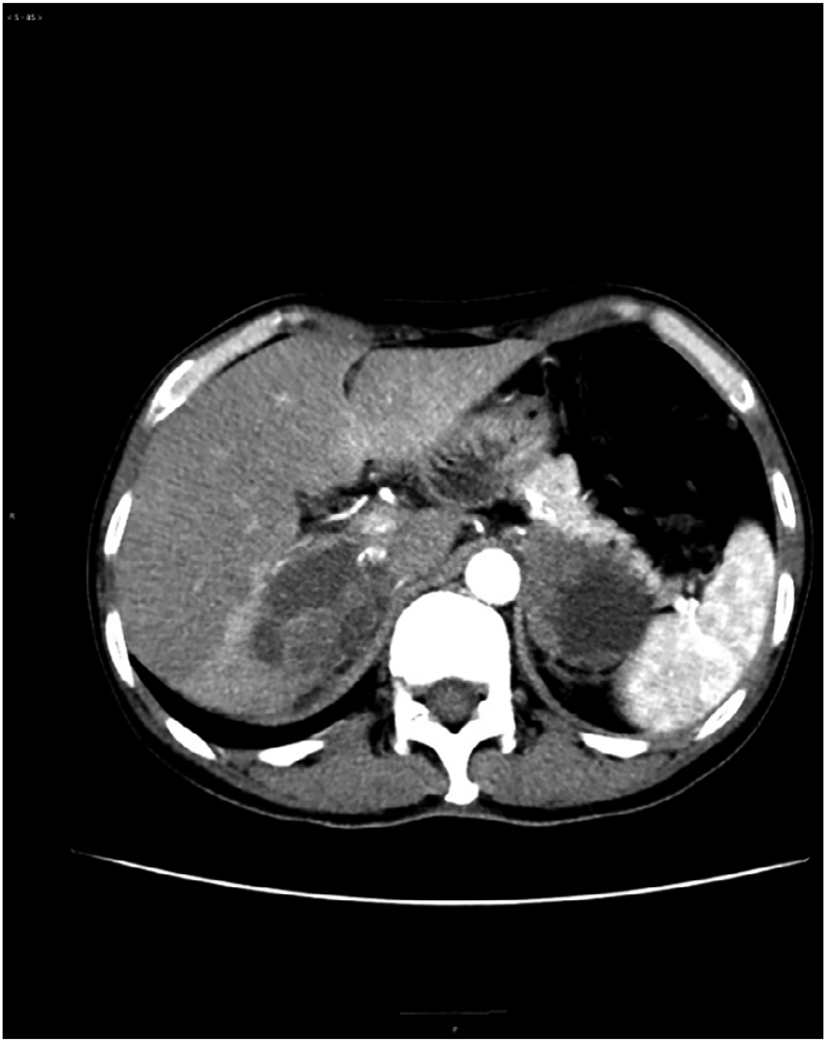
Bilateral adrenal metastases before sintilimab plus bevacizumab treatment (27 June 2018).

A 53‐year‐old man with a history of smoking (30 pack‐years) presented to the hospital for physical examination in March 2017. Chest computed tomography (CT) scan showed space‐occupying lesions of the lower left lung. A CT‐guided percutaneous pneumocentesis biopsy was performed and histological adenocarcinoma was diagnosed. There was no opportunity for surgery at that time because he was already at stage IIIB (cT3N2M0) of diagnosis, with mediastinal lymph node metastasis. He subsequently underwent four courses of chemotherapy (pemetrexed 800 mg D1 plus cisplatin 30 mg D1‐4) and achieved a partial response (PR) based on the Response Evaluation Criteria In Solid Tumors 1.1 (RECIST), during March 2017 to June 2017. Subsequently, primary lung lesions and mediastinal lymph node metastases received intensity modulated radiation therapy (60 Gy/30 F) and the patient also received oral TS‐1 treatment. In October 2017, polymerase chain reaction (PCR) test of the tumor showed *EGFR* exon 19del, but targeted therapy was refused at the time of genotyping. Unfortunately, he was found to have PD (progressive disease) with enlarged primary lung lesions and left hilar nodes in January 2018 after a PFS of 9.6 months receiving first‐line chemotherapy. Based on this, he received gefitinib (250 mg once a day) as the second‐line of treatment. In May 2018, the patient was evaluated and confirmed to have PD following the emergence of right adrenal and liver metastasis, despite a shrunken lung lesion and PFS which had lasted 4.1 months. In consideration of the benefits of gefitinib, treatment with gefitinib was continued until July 2018. On 3 July 2018, the patient underwent rebiopsy of the right adrenal through CT‐guided percutaneous puncture and the histology still indicated adenocarcinoma, and notably PD‐L1 expression 15% + by IHC on Roche Vantana Benchmark IHC (immunohistochemistry) platform with antibody of SP142. Next‐generation sequencing (NGS, Burning Rock, Guangzhou, People's Republic of China) test for a large panel containing 520 oncogenic genes was used for molecular testing of the adrenal biopsy sample, which confirmed the presence of *MET* (MET proto‐oncogene, receptor tyrosine kinase) gene amplification (copy number 10.3) (Fig 1), in addition to the baseline *EGFR* exon 19 del with an allele frequency of 47.5%, and tumor mutation burden (TMB) was calculated (7.9 mutations/Mb). The patient was subsequently switched to bevacizumab (375 mg) combined with gefitinib (250 mg once a day) from July to October 2018. Unfortunately, the disease continued to progress with increases in left adrenal lesions and other metastases (Fig 2).

**Figure 3:**
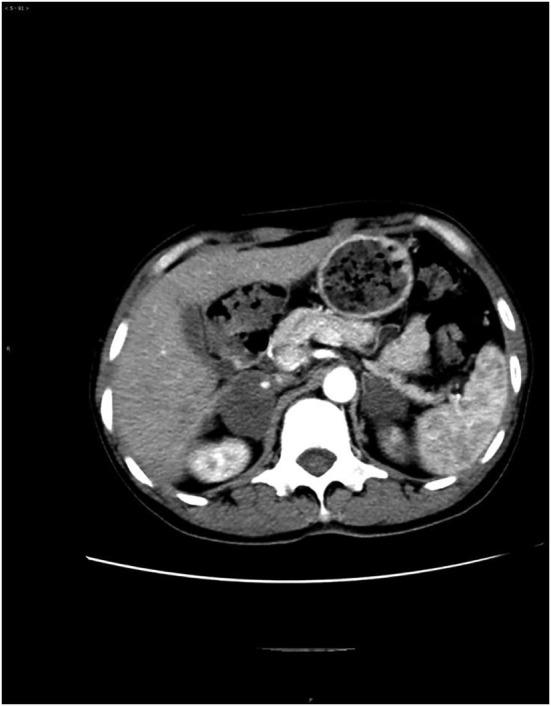
Bilateral adrenal metastases one month after sintilimab plus bevacizumab treatment (20 December 2018).

Due to the poor response to bevacizumab plus gefitinib therapy and PD‐L1 expression 15% + (IHC) on adrenal puncture sample, sintilimab combined with bevacizumab treatment was commenced on 11 November 2018, at a dose of 200 mg and 400 mg, respectively, every three weeks for four times and a PR was obtained after four weeks (Fig 3). During the sintilimab plus bevacizumab treatment, the patient developed grade 3 rashes which can reoccur spontaneously. Myelosuppression, hypertension, diarrhea, or other adverse events were not apparent. Symptoms of dry cough and pain were improved during ICI plus angiogenesis inhibitor treatment. A PFS of 6.0 months was achieved.

**Figure 4:**
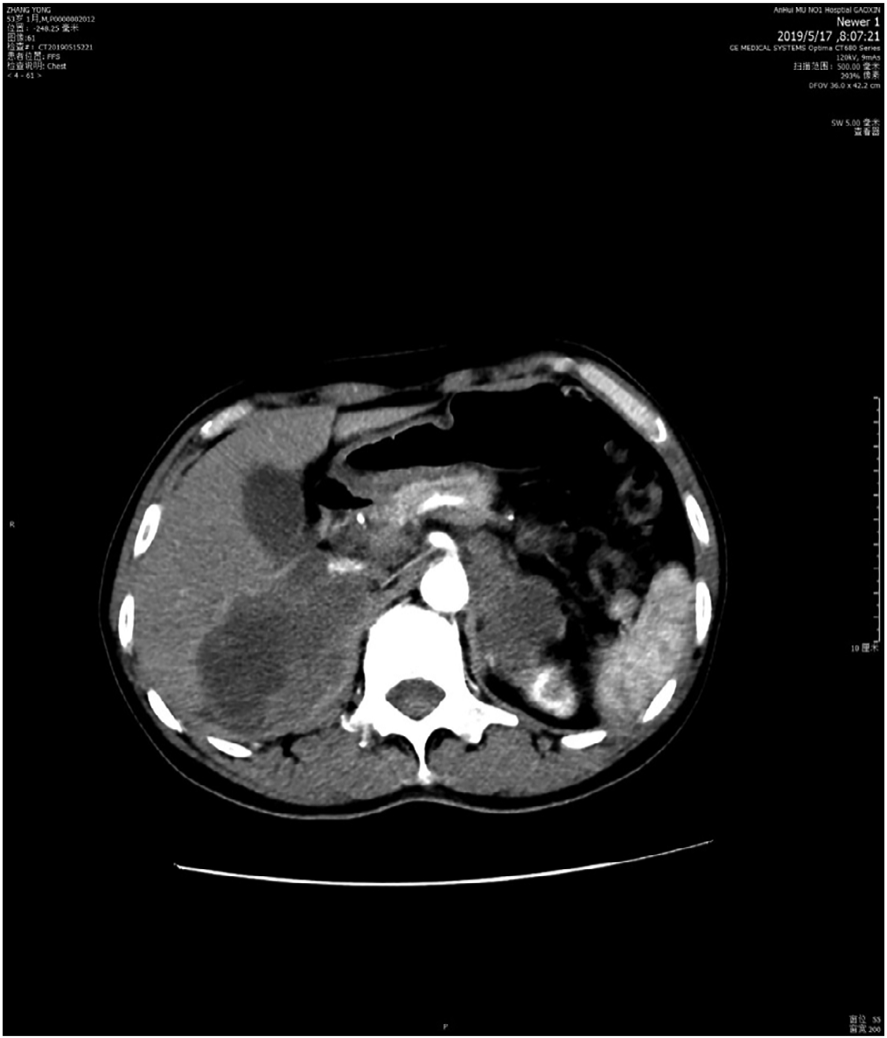
Bilateral adrenal metastases six months after sintilimab plus bevacizumab treatment (15 May 2019).

Unfortunately, thereafter, the patient had progression of bilateral adrenal metastases (Fig 4), his general condition worsened, and he had a dry cough, expectoration and pain. Even if we had attempted to use sintilimab combined with anlotinib or docetaxel, it would not have stopped the disease from deteriorating. The patient died in June 2019.

## Discussion

Over the past decade, many studies have shown that *EGFR* tyrosine kinase inhibitors (TKIs) are preferred for the treatment of advanced NSCLC with *EGFR* mutation.^7^, ^8^ Despite initial efficacy, almost all patients will inevitably develop resistance to these agents.^9^ The molecular mechanisms underlying acquired resistance to *EGFR* TKIs are very complex.^10^, ^11^, ^12^

In this case, after our patient progressed following first‐line treatment, targeted therapy was applied because the patient harbored *EGFR* exon 19 del, but only 4.1 months of PFS was obtained. We considered that the patient's rapid resistance to targeted therapy may have resulted from secondary mutations, or a combination of other mutations. Therefore, molecular testing of the adrenal biopsy sample by NGS was therefore carried out, which confirmed the presence of *MET* gene amplification and *EGFR* exon 19 del. We thought *MET* amplification may have led to acquired resistance to gefitinib, as previous studies have noted that *MET* amplification is a major cause of acquired resistance to the first and third generation *EGFR*‐TKIs, such as erlotinib, gefitinib and osimertinib.^13^, ^14^, ^15^ Crizotinib is effective for patients with *MET* amplification,^16^ but the clinical efficacy of combination therapy of crizotinib and *EGFR*‐TKIs in patients who acquired *MET* amplification during treatment with *EGFR*‐TKIs remains controversial. Based on this, and the poor response to gefitinib, we added bevacizumab, an antiangiogenic drug, when the tumor progressed. However, the response to bevacizumab plus gefitinib therapy was poor.

Recently, ICIs, such as pembrolizumab have been recommended for first‐line treatment in advanced NSCLC patients, whose tumor cell PD‐L1 expression is ≥50% (IHC) and PS (performance status) 0–1. In addition, pembrolizumab and other ICIs combined with platinum or bevacizumab have become one of the first‐line treatment of choice for NSCLC without oncogenic drivers, irrespective of PD‐L1 expression.^17^, ^18^ The emergence of immunotherapy brings a new choice for lung cancer with poor prognosis.

As the PD‐L1 expression was 15% + (IHC) on adrenal puncture sample of this patient, we chose treatment with sintilimab combined with bevacizumab. Sintilimab, a fully human IgG4 monoclonal antibody which can bind to PD‐1 to block the interaction of PD‐1 with PD‐L1 and PD‐L2, has demonstrated its antitumor activity and safety in classical Hodgkin's lymphoma. In phase IA clinical trials in solid tumors it has also been shown to have similar pharmacokinetic characteristics to nivolumab and pembrolizumab.^19^ In our case, the treatment with sintilimab plus bevacizumab conferred six months of PFS in the *EGFR*‐mutant patient with advanced NSCLC who had undergone multiline therapies, which demonstrate its powerful antitumor efficacy. In addition, the toxicity during treatment is mild and controllable. However, it has been suggested that patients with *EGFR* mutations treated with PD‐1/L1 inhibitors (pembrolizumab, nivolumab, and atezolizumab) as second‐line therapy receive very limited benefit from immunotherapy.^5^, ^20^, ^21^ On the other hand, positive results are still being reported following immunotherapy treatment in *EGFR*‐mutated patients. The ATLANTIC study demonstrated that an objective response was achieved in 12.2% patients who had *EGFR*+/*ALK*+ NSCLC with tumor cell PD‐L1 expression ≥25%, treated with durvalumab (anti‐PD‐L1).^22^

Additionally, preclinical and clinical trials have demonstrated synergistic and increased efficacy of antiangiogenic therapy combined with immunotherapy in the treatment of pretreated advanced NSCLC, owing to the immunosuppressive activity of proangiogenic factors,^23^ and it has also been suggested that immunotherapies can be antiangiogenic.^24^ Taken together, it suggests that the combination of these two therapies can act synergistically on the target tumors. Based on this, several trials are currently investigating combinations of angiogenesis inhibitors and ICIs in patients with NSCLC. Preliminary data from such combination therapies are promising. In the study by Reck *et al*. on IMpower150, there was an improved overall survival with ABCP (atezolizumab plus bevacizumab plus carboplatin plus paclitaxel) versus BCP (bevacizumab plus carboplatin plus paclitaxel) observed in patients with sensitizing *EGFR* mutations.^25^ A phase I study evaluated the safety and efficacy of switching to nivolumab maintenance therapy, as monotherapy or combined with bevacizumab in patients with advanced NSCLC who did not progress on first‐line chemotherapy. The results showed improved median PFS of patients treated with nivolumab + bevacizumab compared with nivolumab monotherapy, and the toxicity was well tolerated.^26^ Herbst *et al*. evaluated the safety and tolerability of pembrolizumab combined with ramucirumab in patients with various solid tumors including advanced NSCLC and found that the objective response rate was 30%, while the disease control rate was 85%. The treatment‐related AE was well tolerated.^27^ Based on these results above, in our case, a possible explanation for the achievement of clinical benefit from immunotherapy might be the treatment of sintilimab combined with bevacizumab.

After a PFS of six months, the patient had disease progression without significant change in gene mutation profile, but there was positive downregulation of PD‐L1 (5%) after acquired resistance to immunotherapy. The PD‐L1 expression of tumor tissue may represent a predictive biomarker to ICI immunotherapies and the persistence of PD‐L1 positive tumor tissue in this patient after immunotherapy resistance might reflect a mechanism of immunotherapy escape. In NSCLC, it has been proven that PD‐L1 overexpression is correlated with poor prognosis in patients.^28^ A previous study noted the importance of PD‐L1‐expressing cells promoting dysfunction in tumor‐responding CD8^+^ T cells from pleural effusions in lung cancer patients.^29^ Similar to our results, a previous study has demonstrated that after treatment with nivolumab for six months, patients with PD‐L1 negative circulating tumor cells (CTCs) all obtained a clinical benefit, while patients with PD‐L1 positive CTCs all had PD.^30^

In addition to this, other mechanisms of ICI immunotherapy acquired resistance also include blockade of antigen presentation and new immune escape mutations in tumors (JAK/STAT, B2M).^31^, ^32^ Clinical trials of a combination of immunotherapy with metabolic inhibitors, chemotherapy, and cancer vaccines are underway to provide patients with durable disease control.^33^ Choosing or combining with another immunotherapeutic agent after resistance is also an option, but the preliminary results of clinical trials have so far been unsatisfactory.^34^ In our case, after disease progression following immunotherapy, a combination of sintilimab with cytotoxic chemotherapy, docetaxel and the antiangiogenic drug, anlotinib, were instigated, yet the efficacy was limited.

Although the patient eventually died of disease progression, we have reason to believe that sintilimab plus bevacizumab is a potential option for NSCLC patients with oncogene drivers who progressed in targeted therapy. High expression of PD‐L1 and/or immune cells infiltration might be predictive biomarkers for choosing PD1 inhibition to treat *EGFR*‐mutant NSCLC, and this merits further investigation.
